# Global characterization of microRNAs in *Trichomonas gallinae*

**DOI:** 10.1186/1756-3305-7-99

**Published:** 2014-03-10

**Authors:** Min-Jun Xu, Shen-Ben Qiu, Alasdair J Nisbet, Jing-Hua Fu, Chang-Chun Shao, Xing-Quan Zhu

**Affiliations:** 1 State Key Laboratory of Veterinary Etiological Biology, Key Laboratory of Veterinary Parasitology of Gansu Province, Lanzhou Veterinary Research Institute, Chinese Academy of Agricultural Sciences, Lanzhou, Gansu Province 730046, PR China; 2 Vaccines and Diagnostics, Moredun Research Institute, Pentlands Science Park, Midlothian, EH26 0PZ Scotland, UK; 3 Guangdong Vocational College of Science and Trade, Guangzhou, Guangdong Province 510430, PR China; 4 College of Animal Science, South China Agricultural University, Guangzhou, Guangdong Province 510642, PR China; 5 College of Veterinary Medicine, Yangzhou University, Yangzhou, Jiangsu Province 225009, PR China

**Keywords:** *Trichomonas gallinae*, MicroRNA (miRNA), Quantitative RT-PCR, Gene regulation, Richomonosis

## Abstract

**Background:**

*Trichomonas gallinae* is a protozoan parasite causing trichomonosis in many species of domestic poultry and birds world-wide. microRNAs (miRNAs) are a class of small non-coding RNAs that play key roles in gene regulation. However, no miRNAs have been characterized from *T. gallinae.*

**Methods:**

Here, we investigated the global miRNA profile of this parasite by high throughput sequencing technology, bioinformatics platform analysis and quantitative RT-PCR.

**Results:**

Three miRNA candidates, with typical precursor stem-loop structures, were identified from 11.13 million raw sequencing reads. Three miRNAs, Tga-miR-1, Tga-miR-2 and Tga-miR-3 had no homologues in publically available miRNA databases, suggesting that they may be *T. gallinae-*specific. Tga-miR-2 and Tga-miR-3 occupied only one location each on the reference genome, while Tga-miR-1 was found at 3 locations.

**Conclusions:**

The results of the present study provided a sound basis for the further understanding of gene regulation in this parasite of animal health significance, with the potential to inform the development of novel control reagents and strategies and also inform a more in-depth understanding of the evolution of miRNAs.

## Background

*Trichomonas gallinae* is a flagellated protozoon, which causes avian trichomonosis in many species of domestic poultry and birds, including chickens, pigeons, and parrots. It mainly lives in the anterior digestive tract and causes granulomatous lesions or canker in the host [1]. Different strains of *T. gallinae* vary in their pathogenicity, resulting in a range of clinical outcomes from asymptomatic to lethal infections [2,3]. Certain highly pathogenic strains can affect other organs of birds and cause necrotic foci [4] and peptidases secreted by the parasite were recently reported to have cytopathogenic effects on chicken liver cells [5]. The clinical signs of trichomonosis normally involve anorexia, vomiting, ruffled feathers, weight loss and, in some severe cases, death as a result of starvation [6,7]. Major outbreaks of the parasite cause epidemic mortality, especially breeding populations [8-10]. *T. gallinae* therefore has important welfare and commercial implications for the poultry industry as well as game bird and pigeon breeding and rearing [11]. Trichomonosis has also now been highlighted as a major threat to some endangered wild bird populations, such as the Pink Pigeon in Mauritius [12].

MicroRNAs (miRNAs) are a set of small non-coding RNAs that are now considered as a key mechanism of gene regulation and are essential for the complex life cycles of different parasites [13-16], regulating gene expression at the post-transcriptional level and resulting in post-transcriptional repression [17]. MiRNAs are conserved in metazoans and have been reported in diverse organisms from viruses to mammals [18]. However, despite the veterinary and commercial importance of *T. gallinae* there have been no published studies to date on their miRNAs.

Here we investigated the global miRNA expression profile of *T. gallinae* using a combined platform of next-generation sequencing technology, bioinformatic analysis and real-time quantitative PCR. Due to the similarities between the *Trichomonas* spp., miRNA profile research in *T. gallinae* will shed light on gene regulation studies in other species such as *T. vaginalis*, *T. buccalis* and *T. hominis*, which are medically important parasites in humans.

## Methods

### Ethics statement

The pigeons from which *T. gallinae* were collected, were handled in accordance with good animal practices required by the Animal Ethics Procedures and Guidelines of the People's Republic of China. The present study was approved by the Animal Ethics Committee of Lanzhou Veterinary Research Institute, Chinese Academy of Agricultural Sciences (Approval No. LVRIAEC2011-007).

### Parasites

*T. gallinae* was isolated from pigeons and cultured as described previously with modifications, as follows [19]. *T. gallinae* was collected from the oral cavity of a pigeon with a cotton swab and cultivated *in vitro* in Diamond’s medium supplemented with 10% calf serum and antibiotics (50 IU gentamicin–streptomycin). Cultures were incubated at 36°C for 24 h. The dense cultures were then washed with 0.9% saline for 3 times, and then flash frozen in liquid nitrogen and stored at -80°C. The identity of the cultured parasites was confirmed by sequencing of the ITS of rDNA following PCR amplification with oligonucleotide primers as follows: NC5: 5′-GTAGGTGAACCTGCGGAAGGATCATT-3′; NC2: 5′-TTAGTTTCTTTTCCTCC GCT-3′ (data not shown).

### Total RNA and small RNA isolation

Total RNA of *T. gallinae* was prepared with Trizol reagent according to the manufacturer’s protocol (Invitrogen Co. Ltd). Small RNA was prepared as described previously [20]. Briefly, small RNAs of 20–35 bases in length were isolated from 10 μg total RNA using a 15% TBE-Urea polyacrylamide gel. After adding the 5’ and 3’ adaptors (Illumina Co. Ltd), the fragments were reverse transcribed and then purified with 6% TBE PAGE gel. All gels and kits were purchased from Invitrogen Co. Ltd.

### High-throughput sequencing and computational analysis

Samples were sequenced using a Solexa (Illumina) sequencer. Adaptors and low quality reads were removed from the raw dataset during the base-calling stage. Non-coding RNAs, including rRNA, tRNA, snRNA and snoRNA, were removed by mapping with the Rfam database (http://rfam.sanger.ac.uk/) using BLAST software [21]. Repetitive sequences were removed by searching against the RepeatMasker (http://www.repeatmasker.org) database. Because no publically available genome is currently accessible for *T. gallinae*, the genome of the closely related and well researched species, *T. vaginalis*, from the EuPathDB database (http://eupathdb.org/eupathdb/) was used as a reference genome with reads mapped using SOAP [22]. Mfold was used for the prediction of miRNA candidates [23]. The identified miRNA candidates were then searched against the Sanger miRBase to identify known miRNAs. Potential targets of known miRNAs were predicted with RNAhybrid software [24]. To reduce false-positive results, two extra parameters were applied to the analyzed results: 1) the △△G was set as lower than -25 kcal/mol; 2) P-value was set as ≤ 0.01. The Gene Ontology (GO, http://www.geneontology.org/) database was used for the functional analysis of predicted targets.

### Analysis of novel miRNA expression

Novel miRNAs were analyzed using a modified stem-loop real-time RT-PCR (ABI PRISM^®^ 7300 Sequence Detection System) as described previously [25]. Synthetic lin-4 was used as the endogenous control [26]. The SYBR Green PCR Master Mix was purchased from the TOYOBO Co. Ltd. The amplification cycle conditions were as follows: 95°C for 5 min, followed by 30 cycles of 95°C for 15 s, 65°C for 15 s, and 72°C for 32 s. All reactions were carried out in triplicate. The quantification of each miRNA relative to the lin-4 was calculated using the equation: N = 2^-ΔCt^, ΔCt = Ct_miRNA_-Ct_lin4_[27].

## Results

### Profile characteristics of short RNAs

High throughput sequencing yielded 11.13 million raw reads from the total RNA of *T. gallinae* among which 10.76 million had high quality with no adaptors or polyA regions. Repeat analysis revealed 1,891 sequences as the repeat type LTR:1, 6 sequences as DNA/Maverick:1 and one sequence as DNA/Maverick:0. Therefore, repeat sequences occupied a very small percentage of the high quality reads. rRNA was found to be responsible for a relatively high percentage of the reads (35.71%); and tRNA accounted for 1.84% of the total. Other non-coding RNAs, including snRNA and snoRNA, represented only 0.03% of the reads.

### miRNA profile analysis

**Figure 1:**
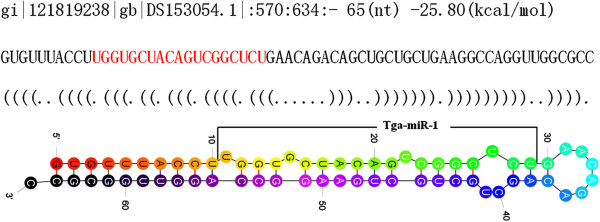
**The stem-loop structure of Tga-miR-1.** The top line indicates gene location, length of precursor, and the energy of the stem-loop structure. The mature miRNA in the precursor is shown in red text and marked in the stem-loop structure.

When mapped onto the *T. vaginalis* genome, 4.47 million reads were successfully mapped. However, of these, only 3 miRNA candidates with precursors having stem-loop structures met the criteria we imposed for miRNA selection (Table 1). These three miRNAs, Tga-miR-1, Tga-miR-2 and Tga-miR-3 had no homologues in the miRBase database. Tga-miR-1 was identified at 3 locations on the reference genome, (scaffolds gi|121819238, gi|121897016 and gi|121907615), while Tga-miR-2 and Tga-miR-3 occupied only one location each on the reference genome. The precursor and stem-loop structure of Tga-miR-1 are shown in Figure 1.

**Table 1:** **miRNA profiles in *Trichomonas gallinae***

**Name**	**Location**	**Arms**	**Mfe**	**Count**	**Sequence**
Tga-miR-1	gi|121819238|:570:634:-	5p	-25.8	6	TGGTGCTACAGTCGGCTCT
Tga-miR-2	gi|121883425|:16027:16113:+	3p	-21.5	19	GAAGAATTGTTGGAAGAA
Tga-miR-3	gi|121907444|:136045:136145:-	5p	-21.2	4	AGAAGGTCTCCTGCCCAC

### Target prediction and function analysis

A total of 60,816 annotated transcripts of *T. vaginalis* were downloaded from the EuPathDB database (http://eupathdb.org/eupathdb/) and used for target prediction of the 3 miRNA candidates. Targets were successfully predicted for the 3 miRNAs with total transcript target numbers being 86 (Tga-miR-1), 2 (Tga-miR-2) and 20 (Tga-miR-3), and with the best matched targets as TVAG_212500, TVAG_158870 and TVAG_068410 for each of the miRNAs respectively. The 3 best matched targets all originated from *T. vaginalis* G3. TVAG_212500 and TVAG_158870 encode hypothetical proteins, while TVAG_068410 encodes a legumain-like cysteine peptidase.

**Figure 2:**
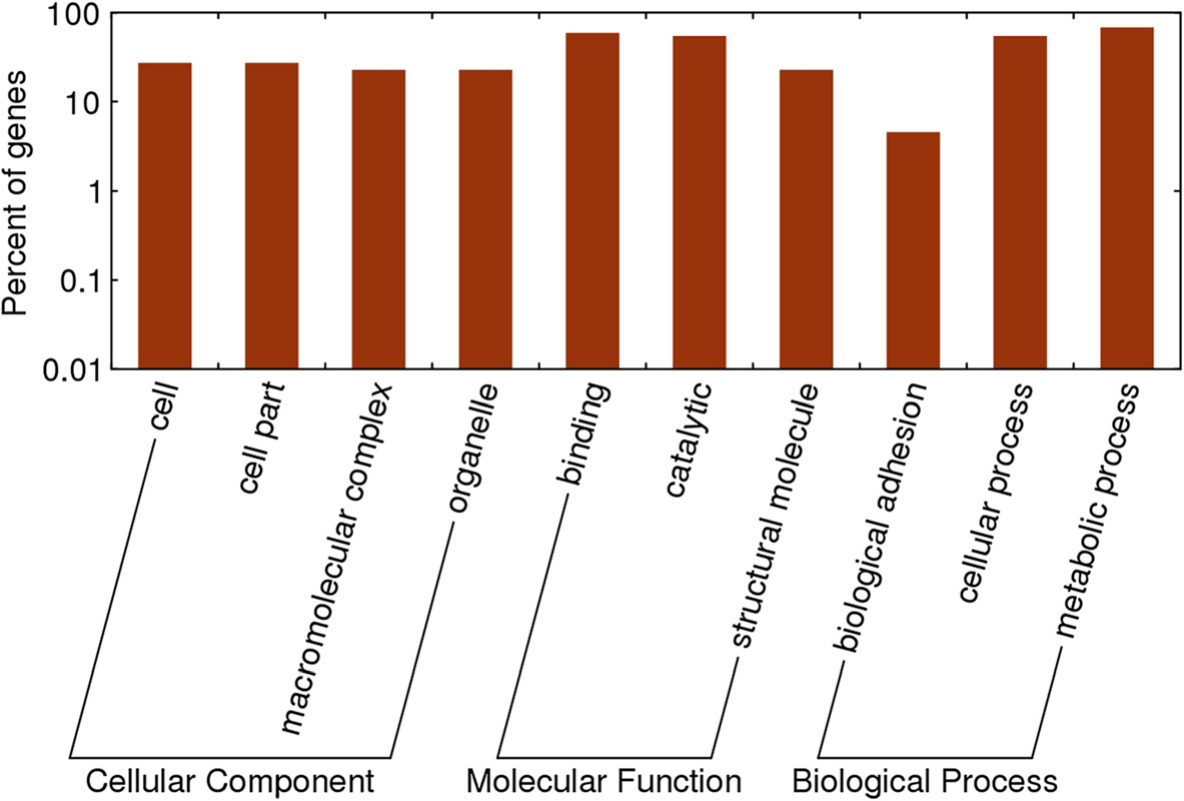
**Enrichment analysis of the predicted targets of *Trichomonas gallinae *** **miRNAs.**

For all the 108 targets, 77 (71.3%) were hypothetical proteins, 4 targets were pe-pgrs proteins and 2 targets, (TVAG_480240, and TVAG_165450), were CAMK family protein kinases. In addition, one transcription activator (TVAG_078540, a “metallothionein-i gene transcription activator”), and one kinase receptor (TVAG_283180, a “leukocyte tyrosine kinase receptor precursor-related protein”) were also found as targets of the 3 miRNAs. Enrichment analysis showed that the molecular function of the targets were biased towards binding and catalytic function (Figure 2).

### miRNAs quantification

Each of the 3 miRNAs could be successfully amplified using qRT-PCR. The relative expression level of Tga-miR-1 (1 ± 0.16) and Tga-miR-2 (1.05 ± 0.12) were similar to the endogenous control. Tga-miR-3 had a significantly higher relative expression level (20.61 ± 1.3) than the other two miRNAs investigated.

## Discussion and conclusions

Avian trichomonosis, caused by the protozoan *T. gallinae*, has been considered as a major disease for numerous avian species. Control by chemotherapy is the main approach to control the disease at present. However, drug resistance coupled with high costs of developing new drugs is still a bottleneck for treatment and prevention of the disease. Therefore, new approaches including immunological, biotechnological and genetic methods are more desirable alternatives [28]. MiRNAs perform a variety of pivotal functions within cells, including regulation of growth, metabolism, development and cell differentiation. They are now considered as biomarkers of parasite invasion, key regulators of gene expression at the post-transcriptional level and potential new tools for disease diagnostics and control [15,29-32].

We herein investigated the global miRNAs of the protozoan *T. gallinae* and 3 miRNA candidates were identified. The number of miRNAs of *T. gallinae* was similar to the miRNAs described by Lin *et al*. [33], who revealed 9 novel miRNAs from *T. vaginalis*, a closely-related parasite. The three novel miRNAs from *T. gallinae* described herein did not possess homologues in miRNAs from *T. vaginalis*, and therefore, the three miRNA candidates identified in the present study may be *T. gallinae*-specific.

Target prediction revealed more than one hundred targets for the 3 miRNAs candidates. However, 71.3% of these targets encoded hypothetical proteins indicating the lack of published confirmatory data for gene prediction and function in *Trichomonas* genomics, in spite of the identification of ~60,000 putative genes in the 170 MB genome of *T. vaginalis*[34]. We identified some miRNA targets as kinases, transcription activators, and receptors, which have fundamental functions in the growth and metabolism of the parasite.

The results of the present study have therefore provided a sound basis for the further understanding of gene regulation in this species, with the potential to inform the development of novel control reagents and strategies and also inform a more in-depth understanding of the evolution of miRNAs.

## Competing interests

The authors declare that they have no competing interests.

## Authors’ contributions

XQZ and MJX conceived and designed the study, and critically revised the manuscript. MJX, SBQ and JHF performed the experiments, analyzed the data and drafted the manuscript. AJN, and CCS helped in study design, study implementation and manuscript revision. All authors read and approved the final manuscript.
